# Security vulnerabilities in healthcare: an analysis of medical devices and software

**DOI:** 10.1007/s11517-023-02912-0

**Published:** 2023-10-04

**Authors:** Carlos M. Mejía-Granda, José L. Fernández-Alemán, Juan M. Carrillo-de-Gea, José A. García-Berná

**Affiliations:** Department of Informatics and Systems, Faculty of Computer Science, University of Murcia, 30100 Murcia, Spain

**Keywords:** Software security, Vulnerability databases, Descriptive study, Software vulnerabilities in health

## Abstract

The integration of IoT in healthcare has introduced vulnerabilities in medical devices and software, posing risks to patient safety and system integrity. This study aims to bridge the research gap and provide valuable insights for addressing healthcare vulnerabilities and their mitigation mechanisms. Software vulnerabilities related to health systems from 2001 to 2022 were collected from the National Vulnerability Database (NVD) systematized by software developed by the researchers and assessed by a medical specialist for their impact on patient well-being. The analysis revealed electronic health records, wireless infusion pumps, endoscope cameras, and radiology information systems as the most vulnerable. In addition, critical vulnerabilities were identified, including poor credential management and hard-coded credentials. The investigation provides some insights into the consequences of vulnerabilities in health software products, projecting future security issues by 2025, offers mitigation suggestions, and highlights trends in attacks on life support and health systems are also provided. The healthcare industry needs significant improvements in protecting medical devices from cyberattacks. Securing communication channels and network schema and adopting secure software practices is necessary. In addition, collaboration, regulatory adherence, and continuous security monitoring are crucial. Industries, researchers, and stakeholders can utilize these findings to enhance security and safeguard patient safety.

**Graphical abstract:**

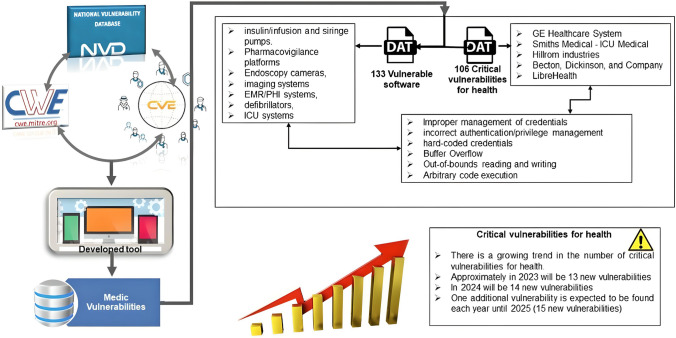

## Introduction

An Internet connection is crucial for information sharing, application updates, and firmware security improvements in the rapidly growing Internet of Things (IoT) field. By 2030, approximately 50 billion IoT devices will be connected to the Internet [1]. In addition, the healthcare sector has embraced IoT (it) wearable devices to enhance patient care and daily life improvements [2]. However, the presence of bugs and vulnerabilities poses (means) a significant risk to the performance and security of these devices. Recently, a substantial increase in vulnerabilities has been recorded, emphasizing the need for robust security measures [3].

Vulnerabilities in computer security refer to weaknesses or errors within a system that attackers can exploit to disrupt its normal functioning [4, 5]. Security vulnerabilities can compromise system operations and endanger accessibility, confidentiality, integrity, and availability [6]. Healthcare businesses have been frequent targets of cyberattacks, with 93 reported incidents between 2013 and 2016 [7]. The resulting healthcare data breaches have been shown to cost an average of $4.35 million by 2022 [8].

Vulnerabilities in networked software applications and devices allow attackers to gain control and carry out illicit activities [9, 10]. Attackers seek to alter the regular operations of systems and devices to achieve persistence, access systems at any time, and propagate the attack to vulnerable adjacent and compatible systems [4, 11]. Security gaps seriously threaten healthcare processes that rely heavily on software automation, such as monitoring blood pressure, electrocardiograms (ECG), oxygen saturation, and body temperature [12]. Additionally, software-assisted breathing systems have played a vital role in managing COVID-19 cases [13].

Malfunctioning software in healthcare settings can pose a significant risk to the patient’s well-being. Historical incidents such as the Therac-25 accelerator accidents [14] and the potential reconfiguration of pacemakers highlight the potential dangers [15]. In addition, a machine delivering medication exhibiting erratic behavior can lead to critical availability issues, potentially resulting in patient harm or even death [16].

Security misconfigurations, lack of adherence to Secure Software Development Life Cycle (SSDLC) methodologies, omission of standards, and poor coding practices contribute to security breaches [17–19]. Many healthcare applications run on outdated operating systems, exacerbating cybersecurity issues. Common vulnerabilities in healthcare stem from cryptographic attacks, cybercrime, denial-of-service attacks, injection exploits, malware, privilege escalation, and web security exploits [20].

To fight against vulnerabilities, organizations such as the Open Web Application Security Project (OWASP) and the National Institute of Standards and Technology (NIST) offer valuable resources. OWASP publishes the Top 10 Web Application Security Risks report guiding mitigating vulnerabilities [21]. NIST’s National Vulnerability Database (NVD) maintains a comprehensive repository of vulnerabilities, including the Common Vulnerabilities and Exposures (CVE) catalog and the associated Common Weakness Enumeration (CWE) [22, 23].

The Common Vulnerability Scoring System (CVSS) classifies the severity of disclosed vulnerabilities [5]. Nowadays, CVSS has undergone three revisions: (i) v1 in 2004 [24], (ii) v2, which includes the CVSS score metric [25], and (iii) v3.1 was published in 2015 for enhancing the process of establishing vulnerability criticality [26]. On the other hand, CWE is a community-developed project to classify security bugs as a list of common software and hardware weaknesses. In addition, it references weakness identification, mitigation, and prevention efforts [27].

Despite the availability of these resources, no metric exists to measure the criticality of life support and medical devices in people’s lives. Additionally, there is a lack of studies focusing on vulnerabilities in healthcare devices and software utilizing official databases like the NVD. This article aims to fill that gap by providing an in-depth analysis of security vulnerabilities in healthcare, specifically targeting medical devices and software from 2001 to 2022, presenting a projection of security issues to rise in 2025 as the cybercriminal tendency and its possible mitigation mechanisms.

## Related work

Mobile device and healthcare network vulnerabilities, classified by Hasan et al. [28] as integrity and confidentiality, privacy, and availability, have been examined. The audit conducted by the authors concludes that the healthcare network demonstrates the availability by resisting Denial of Service (DoS) attacks, Wi-Fi password cracking, and Address Resolution Protocol (ARP) poisoning attacks. However, protection against Wi-Fi password guessing, ARP poisoning, and reverse engineering is essential to ensure the confidentiality and integrity of medical records. Furthermore, the audit reveals that specific medical devices, including pumping machines, remain susceptible to DoS attacks, despite communication with the monitoring system through an SSL-enabled channel. Moreover, the central Electronic Medical Record (EMR) system and the vital sign monitor system of the inspected hospital transmit sensitive login information over Wi-Fi without utilizing the SSL protocol. Therefore, securing communication channels, network schema, medical devices, and technological equipment is crucial for healthcare sector security.

Abouzakhar et al. [29] assess risks and threats associated with IoT security in the healthcare sector. As healthcare systems increasingly adopt distributed cloud computing schemas, new risks emerge in cloud security. These risks include DoS, unauthorized access, ARP poisoning, VM backdoors, hypervisor attacks, rootkit attacks, and VM escape. The primary source of these dangers stems from the lack of interoperability between different IoT protocols and platforms. Disruptions or corruptions in these systems can lead to significant damage or life-threatening risks. Critical IoT systems must maintain a secure and resilient operating environment to mitigate these threats. Cybersecurity for vital IoT systems, such as healthcare systems, presents a challenging and critical issue.

Farhadi et al. [30] conducted a vulnerability scan on an open-source Electronic Health Record (EHR) and medical practice management software OpenEMR. The scan identifies vulnerabilities such as cross-site scripting, file inclusion, HTTP response splitting, control flow attacks, reflection injection, and encryption and decryption issues related to patient history information. The security breaches identified indicate that OpenEMR does not comply with the Health Insurance Portability and Accountability Act (HIPAA) security requirements. The authors recommend patching vulnerable EHR systems and implementing cryptographic methods for persisting and storing PHI data to address these vulnerabilities.

Martinez [31] focuses on determining metrics and vulnerability gaps in medical devices. Despite the Food and Drug Administration (FDA) oversight of these medical devices, a lack of availability and integrity hinders the creation of a relevant list of failure cases. The author emphasizes the need to analyze and classify each medical device connected to the network based on its risk level and complexity. In addition, security breaches should be stored in a centralized record system to enable organization and mitigation. Compromising the integrity and availability of medical devices, such as pacemakers and insulin pumps, poses genuine risks, potentially resulting in injuries or fatalities.

Zakina McGee et al. [32] perform a security analysis of OpenEMR using vulnerability scanning tools. The vulnerabilities identified include cross-site scripting (XSS), SQL injection, and path traversal attacks. The authors successfully mitigate these vulnerabilities after applying scripts and headers in each PHP code file. Early detection of vulnerabilities is essential, and their severity should be prioritized as high, medium, or low-impact vulnerabilities.

Marquez et al. [33] conduct a Systematic Mapping Study (SMS) to detect, classify, and characterize security vulnerabilities in telehealth systems. The study identifies common security challenges within four classifications: attacks, vulnerabilities, weaknesses, and threats. Four security strategies are highlighted: detecting, stopping/mitigating, reacting, and recovering from attacks. In addition, privacy and unsafe data transmission emerge as critical research topics. Finally, software design, requirements, and models are crucial to create safe telehealth systems.

Tervoort et al. [34] conducted a scoping review to offer remedies for reducing cybersecurity dangers brought on by outdated software in medical equipment. The authors identified eighteen solutions linked to medical devices based on intrusion detection or on providing encrypted communication tunnels after they collected and categorized contributions from a selection of papers. They found that security measures are heavily influenced by the sort of medical device they are protecting.

On the other hand, the use of deep learning to predict vulnerabilities in software has been previously studied in the literature. Some examples can be found in works such as in the literature [35]. This paper surveys and reproduces nine deep-learning models for vulnerability detection on the code. It explores model capabilities, training data, and interpretation, revealing the variability and low agreement among models. In another example [36], the paper discusses cyber vulnerability management in a cybersecurity operations center (CSOC) and proposes a novel framework. This one is Deep VULMAN, which uses deep reinforcement learning and integer programming to prioritize and mitigate vulnerabilities. Results show that the framework outperforms current methods in selecting organization-specific vulnerabilities. The use of artificial intelligence (AI) algorithms to provide information security in distrusted networks is proposed in another paper [37]. Key principles of zero trust are presented, and an architecture based on the service-based architecture (SBA) approach is proposed. Our work does not try to predict vulnerabilities from source or binary code. Our work aims to show an overview of vulnerabilities in healthcare software and their consequences from data collected in the NIST vulnerability database. Recommendations are also provided.

## Method

**Fig. 1:**
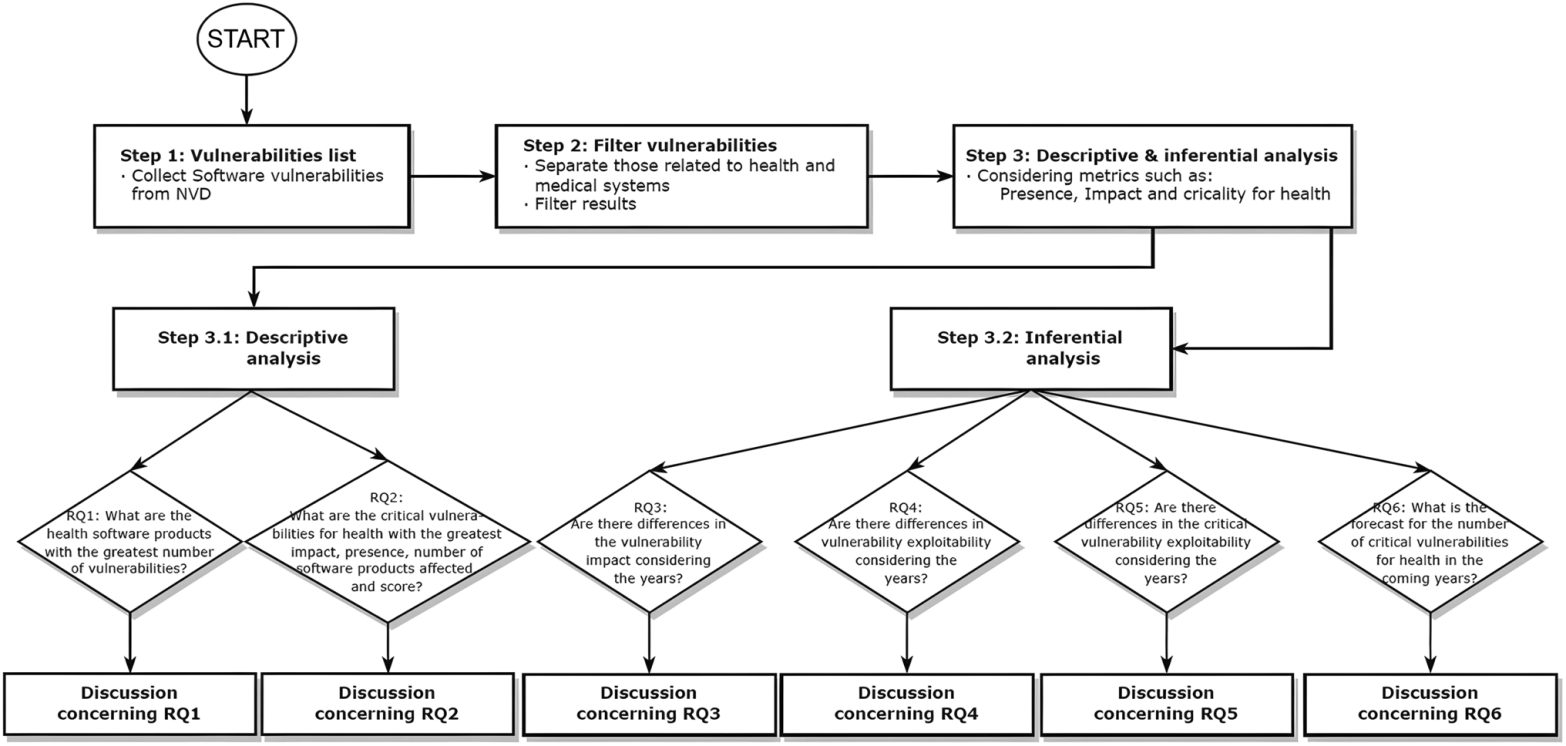
Research flow chart of the method

The method proposed in this paper encompasses three phases. Firstly, all the software vulnerabilities obtained from NVD are compiled. Secondly, the vulnerabilities related to health and medical systems are filtered by using “HEALTH*” and “MEDIC*” as keyword criteria. This search is supported by a data collection tool that the authors developed. Finally, a descriptive and inferential study was carried out, explicitly taking into account the following metrics: presence, impact, and criticality for health per vulnerability. Figure 1 depicts a research flow chart of the method proposed in this paper.

### Data collection

The NVD site is the chosen repository to collect data, given that its list of vulnerabilities in JavaScript Object Notation (JSON) format is periodically updated. CVE and CWE classify the vulnerabilities. Moreover, information is released using the SCAP specifications [26].

Vulnerabilities from 2001 to 2022 were collected with metrics based on MITRE CVSS version 2.0 because version 3.x did not present all vulnerabilities listed in JSON format. Consequently, the parameters considered were the following:

- **CVE_data_meta: id (ID).** Unique vulnerability identifier.
- **Description: (description_data).** Description of each vulnerability.
- **cvssV2: score (baseScore).** According to the CVSS measure, each vulnerability is given a severity rating from 0 to 10. This metric classifies vulnerabilities as moderate if their values are between 0.0 and 3.9, medium if they fall between 4.0 and 6.9, high between 7.0 and 8.9, and critical if they fall between 9.0 and 10.0 [38].
- **cvssV2: accessComplexity (accessComplexity).** Each vulnerability could have different access levels, such as *high, medium, and low*; complexity metrics are described on the NVD official website.
- **cvssV2: authentication (authentication).** Each vulnerability could have a different authentication level, such as *multiple, single,* or *none*. Authentication metrics are described on the NVD official website.
- **cvssV2: confidentiality Impact (confidentialityImpact).** Each vulnerability has a different confidentiality impact, for instance, *none, partial,* or *complete*. Confidentiality impact metrics are described on the NVD official website.
- **cvssV2: integrity Impact (integrityImpact).** Each vulnerability has a different integrity impact: *none, partial,* or *complete*. Integrity impact metrics are described on the NVD official website.
- **cvssV2: availability Impact (availabilityImpact).** Each vulnerability has a different availability impact metric: *none, partial,* or *complete*. All these metrics are described on the NVD official website.
- **problemtype_data: description (value).** The vulnerabilities are grouped using categories. There are over 1000 vulnerability types, all established per the CWE component of SCAP.
- **baseMetricV2: exploitability (exploitabilityScore).** According to the CVSS measure, each vulnerability has an exploitability level that ranges from 0 to 10. According to this metric, a vulnerability is classified as moderate if its value is between 0.0 and 3.9, medium between 4.0 and 6.9, high between 7.0 and 8.9, and critical between 9.0 and 10.0.

In addition, the **criticality for health** parameters was added. A medical specialist analyzed the description of each vulnerability and its impact. If the software or hardware vulnerability could potentially compromise the patient’s life, it will be considered critical for health. Criticality for health metric is expressed by each vulnerability using a binary value: 1 (YES) for critical or 0 (NO) for non-critical matters.

### Obtaining presence and impact per CVE

JSON-formatted data files are annually extracted, which are periodically uploaded and classified by NVD. The calculus of presence metric (${{\varvec{P}}{\varvec{r}}{\varvec{e}}{\varvec{s}}{\varvec{e}}{\varvec{n}}{\varvec{c}}{\varvec{e}}}_{{\varvec{v}}{\varvec{u}}{\varvec{l}}{\varvec{n}}}$ Eq. 1) is the number of products affected by a specific vulnerability versus the number of products affected by all vulnerabilities each year. This comparison expresses the relationship between the participation of a vulnerability concerning all vulnerable health software products in the interval. This metric was taken from Calin et al. [39].

1 $${\mathbf{P}\mathbf{r}\mathbf{e}\mathbf{s}\mathbf{e}\mathbf{n}\mathbf{c}\mathbf{e}}_{\mathbf{v}\mathbf{u}\mathbf{l}\mathbf{n}}=\frac{{\mathrm{Products}}_{\mathrm{vuln}}}{\mathrm{Total Products}}$$

The impact value (${\mathbf{I}\mathbf{m}\mathbf{p}\mathbf{a}\mathbf{c}\mathbf{t}}_{\mathbf{v}\mathbf{u}\mathbf{l}\mathbf{n}}$, Eq. 2) is the relationship between the presence previously calculated and the score found for each vulnerability. This metric was also taken from Calin et al. [39].

2 $${\mathbf{I}\mathbf{m}\mathbf{p}\mathbf{a}\mathbf{c}\mathbf{t}}_{\mathbf{v}\mathbf{u}\mathbf{l}\mathbf{n}}={\mathrm{Presence}}_{\mathrm{vuln}}*{\mathrm{Score}}_{\mathrm{vuln}}$$

The presence and impact calculations are performed for each vulnerability. Furthermore, a JSON file having total presence and impact from NVD-published data feeds is obtained.

### Obtaining presence and impact per each CWE

From collected vulnerabilities, categories are broken down. In this way, a category is associated with its number of vulnerabilities (CVE) and a summary. The average score, which is the average generated from the score meter in vulnerabilities linked to a given category, is the following operation to be carried out.

The presence metric (${\mathbf{P}\mathbf{r}\mathbf{e}\mathbf{s}\mathbf{e}\mathbf{n}\mathbf{c}\mathbf{e}}_{\mathbf{c}\mathbf{a}\mathbf{t}\mathbf{e}\mathbf{g}\mathbf{o}\mathbf{r}\mathbf{y}}$, Eq. 3) is the ratio of the total number of vulnerabilities detected in each category to those found across all CWE.

3 $${\mathbf P\mathbf r\mathbf e\mathbf s\mathbf e\mathbf n\mathbf c\mathbf e}_{\mathbf c\mathbf a\mathbf t\mathbf e\mathbf g\mathbf o\mathbf r\mathbf y}=\frac{{\mathrm{Vulnerabilities}}_{\mathrm{Category}}}{{\mathrm T\mathrm o\mathrm t\mathrm a\mathrm l\;\mathrm v\mathrm u\mathrm l\mathrm n\mathrm e\mathrm r\mathrm a\mathrm b\mathrm i\mathrm l\mathrm i\mathrm t\mathrm i\mathrm e\mathrm s}_{\mathrm{Categories}}}$$

Finally, the impact metric (${\mathbf{I}\mathbf{m}\mathbf{p}\mathbf{a}\mathbf{c}\mathbf{t}}_{\mathbf{c}\mathbf{a}\mathbf{t}\mathbf{e}\mathbf{g}\mathbf{o}\mathbf{r}\mathbf{y}}$, Eq. 4) is the relationship between presence and the average score for each category shown as follows:

4 $${\mathbf{I}\mathbf{m}\mathbf{p}\mathbf{a}\mathbf{c}\mathbf{t}}_{\mathbf{c}\mathbf{a}\mathbf{t}\mathbf{e}\mathbf{g}\mathbf{o}\mathbf{r}\mathbf{y}}={\mathrm{Presence}}_{\mathrm{category}}*{\mathrm{Average\;score}}_{\mathrm{category}}$$

Presence and impact calculations must be performed for each category.

### Analyses of trends for the number of critical vulnerabilities in health

To undertake a trend analysis and determine the number of potential future major health vulnerabilities, the least squares method was employed as a root, as applied by García-Berná et al. [40]. The data between the years 2001 and 2022 were therefore used to calculate a straight line (*Y*, Eq. 5):

5 $$\left(1\right)Y=b \bullet X+a$$

Equation (5) was discovered after solving the following system of equations (Eq. 6, Eq. 7).

6 $$\left(2\right) \sum Y=N\bullet a+b \bullet \sum X$$

7 $$\left(3\right) \sum X\bullet Y=a\bullet \sum X+b \bullet \sum {X}^{2}$$

where *N* is the number of years studied, *Y* denotes the number of major vulnerabilities each year, and *X* represents a conveniently chosen input for the equation to find the coefficients quickly. *X* values are selected so that their sum equals zero, as shown below (Eq. 8):

8 $$\sum\nolimits_{N=2001}^{2022}{X}_{n}=0$$

### Research questions

Once metrics for categories, vulnerabilities, and trend analysis had been defined, the research questions were formulated.

The following questions were answered in this study using descriptive analysis.

- **RQ1.** What are the health software products with the greatest number of vulnerabilities?
- **RQ2.** What are the critical vulnerabilities for health with the greatest impact, presence, number of software products affected, and score?

An inferential analysis will be used to solve the following research questions:

- **RQ3.** Are there differences in the vulnerability impact considering the years?
- **RQ4.** Are there differences in vulnerability exploitability considering the years?
- **RQ5.** Are there differences in the critical vulnerability exploitability considering the years?
- **RQ6.** What is the forecast for the number of critical vulnerabilities for health in the coming years?

IBM SPSS statistical software suite, version 25, was used for data analysis. Before choosing a statistical test over another, some assumptions were considered concerning the data collected in this study. Moreover, when input variables do not follow a normal distribution, a non-parametric test has to be used. Once there are at least three groupings for the variable Years (22 groupings in our study), there are observations of independent studied groups, and the dependent variable has continuous values, the Kruskal–Wallis test is recommended. Additionally, the Kruskal–Wallis H test should be used to compare mean ranks when a researcher analyzes the data morphology and there are abnormal results for several years [41].

## Data collection tool

To repeat this study, simplify information processing, and contribute to this effort, a Java/Maven processing tool was written with the capability to export information in a CSV spreadsheet. The application is hosted at https://github.com/cmejia5486/nistJson.git.

### JSON data feeds

JSON files must be downloaded from the official NIST website with their respective database organized into years and stored in the ../JsonData directory.

#### JSON data feed structure

Each JSON file’s structure consists of a list of vulnerabilities, where each object’s characteristic corresponds to a vulnerability listed in the NVD. A vulnerability object contains its attributes and data collections. The valuable attributes that have been considered for this work are the following.

- **cve: CVE_data_meta:** Contains information related to CVE id per object.
- **cve: Problemtype:** Storage information about the vulnerability’s category as defined by the CVE dictionary.
- **cve: Description:** Contains the description of each vulnerability as a summary.
- **Configurations:** Has relevant information related to vulnerable software for each vulnerability.
- **Impact: baseMetricV2:** Contains the CVSS metrics associated with each vulnerability. The most important metrics are cvssV2: accessVector, cvssV2: accessComplexity, cvssV2: authentication, cvssV2: confidentialityImpact, cvssV2: integrityImpact, cvssV2: availabilityImpact, and cvssV2: baseScore
- **Impact: exploitabilityScore:** References to exploitability metric per each vulnerability.

#### Extra data feed files

The description associated with each category is located in the file ../CweDefinitions/summary.txt. Moreover, there is a file to store criticality parameters for health in ../Health/metrics.csv. In addition, the ../JsonData/Total.json data file was constructed by the union of each data feed file, keeping the JSON structure.

### Source code explanation

**Fig. 2:**
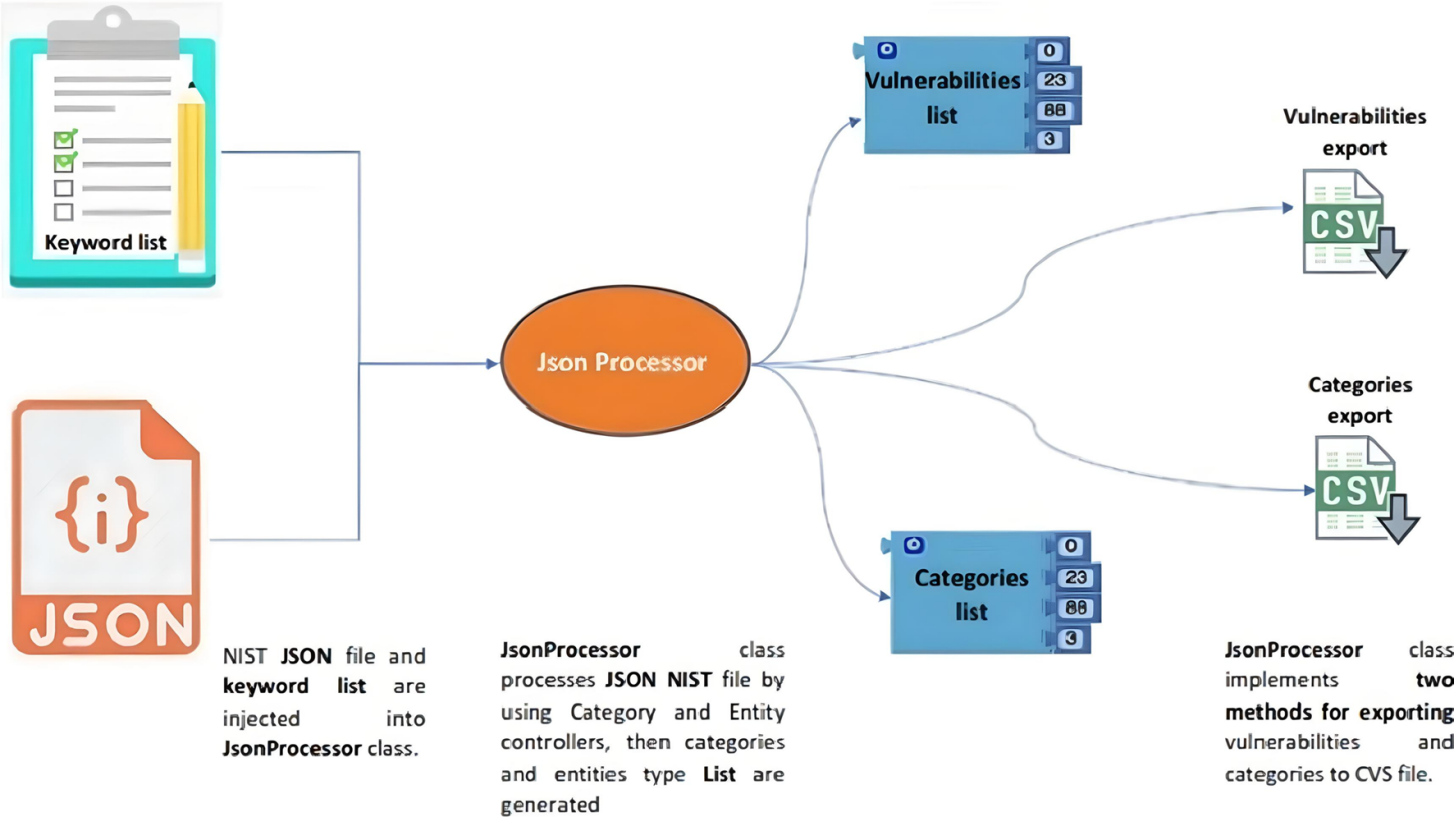
Data collection tool diagram

Documentation referring to the Java methods and classes is in the directory ../target/site/apidocs. Figure 2 illustrates the tool’s usual execution path. From the JSON file and keywords given for searching, the researcher obtained a list of vulnerabilities, categories, and exclusion vulnerabilities (CVE) after a one-by-one analysis of the vulnerabilities extracted by the tool to exclude false positives. Each vulnerability is analyzed and removed if unrelated to the subject of study. In this work, the file ./Exclusions/exclusions.txt contains 88 CVE items to be excluded, their identifier, and the reason for excluding them from the test according to a medical specialist criterion consequently, the class ../src/nist.main will create summarized CSV files for being analyzed by the researcher with all the specific data presented in 3.1 section. (Fig. 4).

## Results

In this section, we outline the results of the proposed RQs. Appendices will be included in the repository to provide graphical support for answering each question. The repository is available at https://github.com/cmejia5486/p1_appendixes.git

### Descriptive analysis

Information on vulnerabilities and categories was collected in January 2023. The total number of vulnerabilities related to medical vulnerabilities in software and devices was 201 from 2001 to 2022.

#### Vulnerabilities analysis

- RQ1. What are the health software products with the greatest number of vulnerabilities?

The top ten health software products with the greatest quantity of vulnerabilities are detailed in Table 1. The first fifty software products with the greatest number of vulnerabilities are presented in Appendix A Fig. 4.

**Table 1:** Top ten health software products with the greatest number of vulnerabilities between 2001 and 2022

*N*	Software	N. CVE	Vendor
1	librehealth_ehr	21	LibreHealth
2	gehealthcare:centricity	11	General Electric
3	oracle:argus_safety	10	Oracle Corporation
4	artmedic_webdesign:artmedic	9	ArtMedic
5	swisslog-healthcare:hmi-3_control_panel	8	Swisslog
6	smiths-medical:medfusion	8	smiths-medical
7	oracle:industry_applications	7	Oracle Corporation
8	gehealthcare:discovery	6	General Electric
9	medicomp:medcin_engine	5	medicomp
10	ishekar:endoscope_camera	5	Ishekar

- RQ2. What are the critical vulnerabilities for health with the greatest impact, presence, number of software products affected, and score?

There were 106 of the 201 vulnerabilities considered critical for health. The top ten vulnerabilities with the greatest impact are detailed in Table 2. All health vulnerabilities ranked from highest to lowest impact are shown in Appendix B Fig. 5a.

**Table 2:** Top ten criticality for health vulnerabilities with the greatest impact between 2001 and 2022

*N*	Vulnerability	Impact	Vendor	Year
1	CVE-2022–22766	0.186	Becton, Dickinson, and Company (BD)	2022
2	CVE-2021–27410	0.138	Hillrom	2021
3	CVE-2018–14786	0.111	Becton, Dickinson, and Company (BD)	2018
4	CVE-2018–4846	0.111	Siemens AG	
5	CVE-2021–27408	0.092	Hillrom	2021
6	CVE-2021–32025	0.080	Blackberry	
7	CVE-2018–4845	0.072	Siemens AG	2018
8	CVE-2017–14006	0.069	GE Healthcare	2017
9	CVE-2016–8355	0.066	Smiths Medical – ICU Medical	2016
10	CVE-2021–22156	0.063	Blackberry	2021

The top ten vulnerabilities with the greatest presence are detailed in Table 3. The complete list of health vulnerabilities ordered from highest to lowest presence is shown in Appendix B Fig. 5b.

**Table 3:** Top ten criticality for health vulnerabilities with the greatest presence between 2001 and 2022

*N*	Vulnerability	Presence	Vendor	Year
1	CVE-2022–22766	0.088	Becton, Dickinson, and Company (BD)	2022
2	CVE-2021–27410	0.018	Hillrom	2021
3	CVE-2021–27408	0.018		
4	CVE-2018–14786	0.015	Becton, Dickinson, and Company (BD)	2018
5	CVE-2018–4846	0.011	Siemens AG	
6	CVE-2021–32025	0.011	Blackberry	2021
7	CVE-2018–4845	0.011	Siemens AG	2018
8	CVE-2017–14006	0.009	GE Healthcare	2017
9	CVE-2021–22156	0.009	Blackberry	2021
10	CVE-2016–8355	0.007	Smiths Medical – ICU Medical	2016

The top ten health vulnerabilities grouped by the number of software products affected are detailed in Table 4. All health vulnerabilities ranked from highest to lowest number of software products are shown in Appendix B Fig. 5c.

**Table 4:** Top ten criticality for health vulnerabilities with the greatest number of software products between 2001 and 2022

*N*	Vulnerability	N. Software	Vendor	Year
1	CVE-2022–22766	48	Becton, Dickinson, and Company (BD)	2022
2	CVE-2021–27410	10	Hillrom	2021
3	CVE-2021–27408	10		2021
4	CVE-2018–14786	8	Becton, Dickinson, and Company (BD)	2018
5	CVE-2018–4846	6	Siemens AG	
6	CVE-2021–32025	6	Blackberry	2021
7	CVE-2018–4845	6	Siemens AG	2018
8	CVE-2017–14006	5	GE Healthcare	2017
9	CVE-2021–22156	5	Blackberry	2021
10	CVE-2016–8355	4	Smiths Medical – ICU Medical	2016

The top ten health vulnerabilities sorted in order from highest to lowest score are detailed in Table 5. The complete list is shown in Appendix B Fig. 5d.

**Table 5:** Top ten criticality for health vulnerabilities with the greatest score between 2001 and 2022

*N*	Vulnerability	Score	Vendor	Year
1	CVE-2014–5406	9.3	Hospira	2014
2	CVE-2019–11687	9.3	Nema	2019
3	CVE-2016–8355	9.0	Smiths Medical – ICU Medical	2016
4	CVE-2020–11439	9.0	LibreHealth	2020
5	CVE-2022–31496	9.0		2022
6	CVE-2021–37166	7.8	Swisslog-Healthcare	2021
7	CVE-2021–27410	7.5	Hillrom	
8	CVE-2018–14786	7.5	Becton, Dickinson, and Company (BD)	2018
9	CVE-2017–14006	7.5	GE Healthcare	2017
10	CVE-2017–12726	7.5	Smiths Medical – ICU Medical	

Criticality for health vulnerabilities is highly influenced by (i) improper management of credentials (user/password), authentication, or incorrect privilege management. For example, the lack of complete proof that an actor’s claim of having a particular identity is valid prevents the software from correctly assigning, modifying, tracking, or checking rights for the actor, leading to unexpected consequences and an absence of control; (ii) out-of-bounds reading and writing which causes data corruption, crash, or improper code execution; (iii) hard-coded credentials usually allow an attacker to bypass the authentication and communicate with external devices or systems, even, before performing crucial functionality that needs a user identification that can be verified or that also uses a lot of resources, the software frequently does not do any authentication.

Also, corrupt data, arbitrary code execution, system crash/stoppage due to out-of-confines writing, or improper restriction of operations inside the bounds of a memory buffer are additional effects of the vulnerabilities examined in this study. This type of vulnerability is severe because this study’s analyzed devices/software is related to medical support and patient life care.

Conversely, the primary affected industries were as follows:

- GE Healthcare systems, because of sensitive information exposure and poor management of credentials, an attacker can access sensitive personal health information (PHI). Medical information could be used for perpetuating cybercrimes by using false identities with complete names, patient numbers, social security, etc.
- Smiths Medical – ICU Medical software presents a loss of confidentiality and integrity because of incorrect input handling and poor management of credentials. Due to corrupted information managed by the medication safety software, a patient’s medicine can be mistakenly administered and finally threaten their health.
- Hillrom Industries – Welch Allyn medical device management tools are prone to data corruption and malicious code execution due to buffer overflow. Therefore, patient health supported by pressure gauges, visual control devices, cardiac meters, and vital monitoring could be compromised.
- Becton, Dickinson, and Company (BD), with Alaris medical devices product, compromise confidentiality, integrity, and availability due to a weakness related to improper authentication. As a result, patients’ health could be critically compromised since a system that controls the supply of the medicine delivered by syringes is undermined.

### Hypothesis test

The data was examined once global data for health/medical scope between 2001 and 2022 regarding the impact, presence, exploitability score, products affected per vulnerabilities, and categories were presented. As a result, some behavior patterns were identified that led us to propose the following research questions:

- RQ3. Are there differences in the vulnerability impact considering the years?

In the statistical analysis, the independent variable was the year, and the dependent variable was the vulnerability impact. After comparing the average ranks of the impact of the vulnerabilities between 2001 and 2022 using the Kruskal–Wallis test, taking into account a mean of 0.05 and a standard deviation of 0.261, statistically significant differences were found (*χ*^2^(2) = 47.765, *p* = 0.001). In addition, six pairings of years with a *p*-value under 0.05 showed statistically significant differences in the post hoc contrasts presented in Table 6.

**Table 6:** Statistically significant differences in post hoc contrast for impact between 2001 and 2022

*N*	Years couple	Mean in years	Contrast statistic	*P*. sig
1	2022–**2017**	0.019–**0.035**	74.204	0.000
2	2022–**2013**	0.019–**0.056**	92.669	0.000
3	2022–**2010**	0.019–**0.033**	83.558	0.002
4	2020–**2017**	0.019–**0.035**	49.979	0.003
5	2020–**2013**	0.019–**0.056**	68.444	0.003
6	2015–**2013**	0.019–**0.056**	75.899	0.004

The years in which a major impact of vulnerabilities was produced is highlighted in boldface

Consequently, the impact metric in 2013 had the highest value, with an average of 0.056, and presented statistically significant differences from the mean of the other years, according to the values shown in Table 6.

- RQ4. Are there differences in vulnerability exploitability considering the years?

In the statistical analysis, the independent variable was the year and the dependent the vulnerability exploitability. Comparing the mean ranks of the exploitability of the vulnerabilities between 2001 and 2022 using the Kruskal–Wallis test, statistically significant differences were found (*χ*^2^(2) = 38.461, *p* = 0.011), taking into account a mean of 8.17 and a standard deviation of 2.095. In the post hoc contrasts (see Table 7), statistically significant differences were found in 5 pairs of years (*p*-value less than 0.05).

**Table 7:** Statistically significant differences in exploitability between 2001 and 2022

*N*	Years couple	Mean in years	Contrast statistic	*P*. sig
1	2014–**2010**	6.988 – **10**	86.353	0.001
2	2020–**2010**	7.457 – **10**	79.435	0.002
3	2016–**2010**	6.067 – **10**	122.333	0.002
4	2013–**2010**	6.711 – **10**	88.056	0.003
5	2016–**2012**	6.067 – **10**	122.333	0.004

The years in which a major impact of vulnerabilities was produced is highlighted in boldface

Consequently, according to our findings, the exploitability metric in 2010 and 2012 has the highest value with a mean of 10. It has statistically significant differences from the mean of the other years, according to the values shown in Table 7.

- RQ5. Are there differences in the critical vulnerability exploitability considering the years?

In the statistical analysis, the independent variable was the year, and the dependent variable, the critical vulnerability exploitability. A total of 106 out of the 201 software vulnerabilities are critical for health. Applying the Kruskal–Wallis test to compare its mean ranks between 2001 and 2022, statistically significant differences were found (*χ*^2^(2) = 37.347, *p* = 0.007), taking into account a mean of 8.381 and a standard deviation of 2.175. In the post hoc contrasts (see Table 8), statistically significant variations were discovered in six pairs of years (*p*-value less than 0.05).

**Table 8:** Statistically significant differences for exploitability in vulnerabilities critical for health between 2001 and 2022

*N*	Years couple	Mean in years	Contrast statistic	*P*. sig
1	2020–**2010**	6.839 – **10.000**	46.538	0.002
2	2016–**2010**	6.067 – **10.000**	63.833	0.002
3	2020–**2021**	6.839 – **9.269**	-34.385	0.002
4	2022–**2010**	7.615– **10.000**	44.615	0.003
5	2022–**2021**	7.615 – **9.269**	32.462	0.004
6	2016–**2012**	6.067 – **10.000**	63.833	0.004

The years in which a major impact of vulnerabilities was produced is highlighted in boldface

Consequently, the metric of the exploitability vulnerabilities critical for health in 2010 and 2012 have the highest value with an average of 10 and presents statistically significant differences from the mean of the other years, according to the values shown in Table 8.

- RQ6.What is the forecast for the number of critical vulnerabilities for health in the coming years?

After using the least squares method to estimate the number of critical vulnerabilities for health that may emerge in the future (until 2024), the straight line calculated and shown in Table 9 predicts that one more vulnerability will be developed each year compared to the previous one.

**Table 9:** Number of critical vulnerabilities for health projected to 2024

Year	The actual number of critical vulnerabilities for health (*Y*)	Input (*X*)	*X*·*Y*	${X}^{2}$	Number of critical vulnerabilities for health forecast
2025		29			15.007
2024		27			14.320
2023		25			13.634
2022	13	23			12.947
2021	13	21	273	441	12.260
2020	13	19	247	361	11.573
2019	4	17	221	289	10.886
2018	8	15	60	225	10.199
2017	19	13	104	169	9.512
2016	3	11	209	121	8.825
2015	1	9	27	81	8.137
2014	5	7	7	49	7.452
2013	4	5	25	25	6.765
2012	4	3	12	9	6.078
2011	5	1	4	1	5.391
2010	5	− 1	− 5	1	4.704
2009	1	− 3	− 15	9	4.017
2008	0	− 5	− 5	25	3.330
2007	2	− 7	0	49	2.644
2006	1	− 9	− 18	81	1.957
2005	0	− 11	− 11	121	1.270
2004	1	− 13	0	169	0.583
2003	1	− 15	− 15	225	− 0.104
2002	2	− 17	− 17	289	− 0.791
2001	1	− 19	− 38	361	− 1.478

**Fig. 3:**
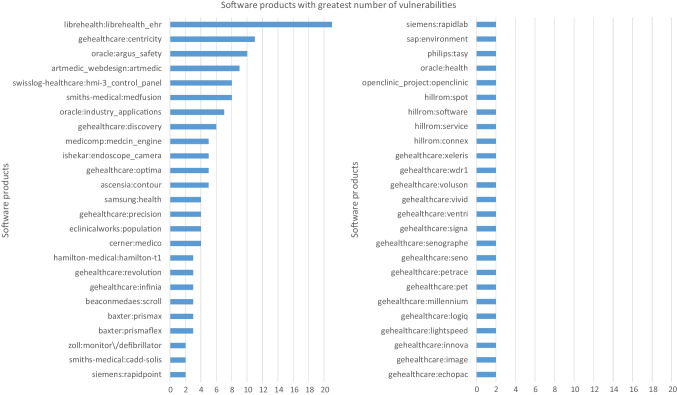
The first fifty software products with the greatest number of vulnerabilities

**Fig. 4:**
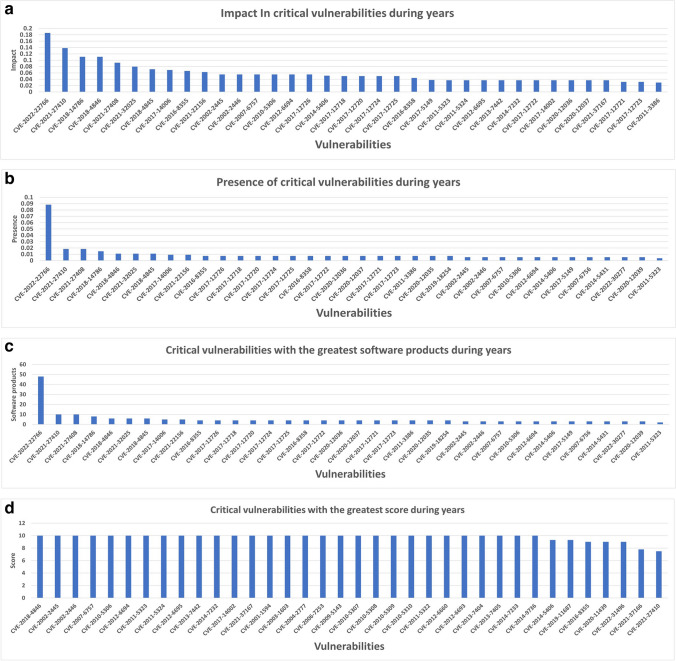
Tendency analysis graph of the number of critical vulnerabilities for health

**Fig. 5:**
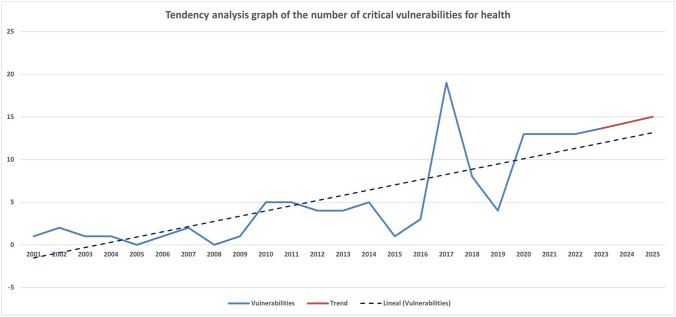
**a** Health vulnerabilities ranked from highest to lowest impact from 2001 to 2022. **b** Health vulnerabilities ordered from highest to lowest presence from 2001 to 2022. **c** Health vulnerabilities ranked from highest to lowest number of software products from 2001 to 2022. **d** Health vulnerabilities sorted from highest to lowest score from 2001 to 2022

Figure 3 shows that the tendency of the number of critical vulnerabilities for health per year versus the actual number per year is expected to grow.

## Discussion

Results from Section 5 were assessed to determine the study’s principal conclusions.

- RQ1. What are the health software products with the greatest number of vulnerabilities?

The study’s findings indicate that the health software solutions with the most vulnerabilities are EHR, wireless infusion pumps, endoscope cameras, and radiology information systems. However, despite having security techniques for ensuring medical devices, many vulnerabilities are still present [42, 43]. Considering that health software products support more of the activities in hospitals, several studies agree with us in making it secure against cyberattacks before use in patients to avoid potential health threats [44, 45].

- RQ2. What are the critical vulnerabilities for health with the greatest impact, presence, number of software products affected, and score?

The critical vulnerabilities for health with the greatest impact, presence, number of software products affected, and score are majority caused by (i) poor management of credentials enabling unintended actions and lack of control; (ii) buffer out-of-bounds causing data corruption and improper code execution; and (iii) hard-coded credentials which bypass the authentication in software functions. These weaknesses coincide with the security challenges in health care addressed by Randolph C. Barrows et al. [46]. Unfortunately, encrypted passwords could be present in all programming languages and all operating platforms; this is why it is recommended to mitigate this vulnerability by using [47]:

- *Design (for default accounts):* Use an “initial login” mode that necessitates the user entering a distinct strong password rather than hard-coding a default username and password for first-time logins.
- *Design (for front-end to back-end connections):* There are three potential answers, but none is perfect. **(i) First recommendation** calls for randomly generated passwords that are updated automatically and that a system administrator must enter at predetermined intervals. These passwords will be stored in memory and are only effective during the designated periods. **(ii) Second**, rather than allowing unlimited access, the passwords used should be restricted at the back end to only executing operations required for the front end. **(iii) Finally**, to avoid replay-style assaults, the messages sent should be tagged, verified, and summarized with time-sensitive values.

Moreover, results exposed in RQ2 also revealed that the primary industries involved in criticality for health care are GE Healthcare systems, Smiths Medical – ICU Medical software (syringe infusion pumps), Hillrom industries (Welch Allyn medical device), Becton, Dickinson, and Company (Alaris medical devices), Siemens AG and Blackberry, meaning that health care field continues being through security breaches. For example, syringe infusion pumps are still being attacked by FTP server exploitation [48].

- RQ3. Are there differences in the vulnerability impact considering the years?

Despite having an approval and regulatory process for medical devices by the FDA since 1976 [49], the year with the highest impact mean value was 2013. Insulin pumps, X-ray systems, blood refrigeration units, and so on are still vulnerable and are targets for cybercriminals [7]. Patient health information and social security numbers are highly profitable on the dark web, sold 10 to 20 times more than other data types [50, 51]. Moreover, medical data stolen by cyber criminals continue causing negative impacts with numerous losses in patients’ treatment plans and hospital operations [52]. Implications for software and healthcare devices can be mitigated by (i) eliciting secure software requirements, (ii) implementing vulnerability detection before the deployment, and (iii) testing and reviewing medical devices during all phases by manufacturers, framed in current regulations [9].

- RQ4. Are there differences in vulnerability exploitability considering the years?

The highest exploitability mean was in the years 2010 and 2012. Our findings align with an analysis conducted by the Kenna Security of more than 100,000 vulnerabilities disclosed since 2011 [53]. However, the percentage of exploitable vulnerabilities has dropped over the past years. Exploitability can be due to poor and inadequate infrastructure [54], lack of political will [55], low technology acceptance [56], minimal research [55], limited connectivity [57], inadequate human resources [58, 59], lack of policies and legal framework [60], and using legacy operating systems such as Windows XP [61]. However, the weakest link in cybersecurity and security failures across many information technology domains lies in human errors, which must be minimized and controlled [62]. Other good practices for mitigating exploitability are (i) protecting health information against privileged; (ii) reducing to the minimum the number and type of privileged accounts [63]; (iii) periodically changing management, credential updating, logging, and monitoring according to best ITIL practices [64]; and (iv) implementing ongoing life cycle processes and continued safety post-market monitoring by manufacturers according to FDA [65]. The developers and security analysts must prioritize managing the vulnerabilities based mainly on two criteria: ease of being exploited by attackers and severity of the damage caused to the system.

- RQ5. Are there differences in the critical vulnerability exploitability considering the years?

In contrast, with the general trend, the exploitability of critical vulnerabilities is increasing, with a peak of 10 achieved in 2010 and 2012. Considering that vulnerabilities critical to health are potentially dangerous for patients’ health, assessing the exploitability of vulnerabilities is an essential concern for security defenders, developers, and manufacturers. However, finding exploitable states in software and devices takes a long time [66, 67]. Forecasting vulnerability exploitability is essential to previous decisions and efforts before being exploited because, unfortunately, it is difficult to patch all vulnerabilities [68]. Providentially, machine learning predicts exploitable vulnerabilities before discovering them [69].

- RQ6. What is the forecast for the number of critical vulnerabilities for health in the coming years?

Security gaps and vulnerabilities have been boosted since 2010 [61]. Once holes are published, their exploitability magnitude increases five times [70]. Only in 2019, software vulnerabilities have increased by more than 130.000, according to open-source databases [67]. By our results is expected that at least one vulnerability critical for health increase each year until 2025.

## Conclusions and further work

This research identified vulnerable industries and studied security breaches affecting electronic devices and software used in critical patient support. It also examined trends and techniques used by cybercriminals to exploit security gaps in eHealth systems. The findings provide recommendations for healthcare software industries, researchers, and users to develop secure software solutions and robust applications and implement security patches or enhancements.

The analysis revealed that the user remains the weakest link in the security chain. There are persistent vulnerabilities, such as poor credential management, sensitive information exposure, and incorrect authentication at well-known companies that produce critical health devices. This upward trend of security breaches in healthcare devices and software is expected to continue.

Implementing recommended security measures and adopting a Secure Software Development Life Cycle (SSDLC) can improve system and device security by mitigating vulnerabilities.

Future work involves the creation of a catalog of safety requirements aligned with regulations, best practices, and standards to ensure quality control in the development of healthcare systems and devices. In addition, a continuous improvement audit method will also be developed to control the vulnerabilities identified during the production stage and before the market launch.
